# Lessons Learned from Air Quality Assessments in Communities Living near Municipal Solid Waste Landfills

**DOI:** 10.3390/ijerph22111732

**Published:** 2025-11-15

**Authors:** Custodio Muianga, John Wilhelmi, Jennifer Przybyla, Melissa Smith, Gregory M. Zarus

**Affiliations:** 1 Office of Innovation and Analytics (OIA), Agency for Toxic Substances and Disease Registry (ATSDR), Center for Disease Control and Prevention (CDC), Department of Health and Human Services (DHHS), Atlanta, GA 30341, USAzee7@cdc.gov (M.S.); gmzarus@gmail.com (G.M.Z.); 2 Eastern Research Group, Inc., Boston, MA 02109, USA; john.wilhelmi@erg.com

**Keywords:** emissions, landfills, community actions

## Abstract

Over 292 million tons of municipal solid waste (MSW) are generated annually in the United States, with more than half disposed of in landfills. Municipal solid waste landfills (MSWLFs) are stationary sources of air pollution and potential health risks for nearby communities. The Agency for Toxic Substances and Disease Registry (ATSDR) has completed over 300 public health assessments (PHAs) and related investigations at MSWLFs and open dumps since the 1980s. This paper reviews the ATSDR’s evaluations of air pathway concerns at 125 MSWLF sites assessed between 1988 and early 2025, with many being evaluated during the 1990s. Most sites were located in the Midwest and Northeast, and only 25% remained active. The ATSDR found no air-related public health hazard at 86% of sites. At sites where hazards were identified, common issues included elevated outdoor or indoor toxicants (e.g., hydrogen sulfide, benzene, trichloroethylene, and mercury) and unsafe methane accumulations. Contributing factors included older site designs, inadequate gas-collection, subsurface fires, and distance from nearby residences. Corrective actions effectively reduced exposures at the affected sites. Results suggest that well-located and maintained landfills minimize public health hazards, while aging or poorly managed sites pose risks. Continued monitoring and research are warranted as waste management shifts toward reducing, reusing, recycling, composting, and energy-recovery technologies to improve efficiency, advance technologies, and address systemic public health challenges.

## 1. Introduction

The Agency for Toxic Substances and Disease Registry (ATSDR) protects communities from the harmful effects of hazardous substances in the environment. The ATSDR does this by investigating environmental health threats in communities and taking action to reduce harmful exposures and their health consequences. For more than 40 years, the ATSDR has applied this mission to a broad array of anthropogenic and natural sources of environmental contamination [1,2]. The sources include municipal solid waste landfills (MSWLFs) and open dumps.

According to the United States (U.S.) Environmental Protection Agency (EPA), an MSWLF is a “discrete area of land or excavation that receives household waste” [1,2]. MSWLFs may receive other types of waste, such as non-hazardous commercial and industrial solid waste. MSWLFs are regulated under Subtitle D of the Resource Conservation and Recovery Act (RCRA) [2]. Each state is responsible for ensuring that all MSWLFs within its jurisdiction meet the federal criteria for operation. The states may also set more stringent requirements. Approximately 1900 active MSWLFs currently operate in the United States [1,2,3]. In addition, nationwide, several thousand MSWLFs are closed. Millions of Americans live near these waste sites and may inhale site-related air pollution. MSWLFs pose environmental contamination concerns and are disproportionately located in communities of low-income populations [2,3,4].

Subtitle D of the RCRA regulations prohibits the open dumping of solid waste, with a few exceptions. However, before 1979, open dumping was a common practice throughout the United States. Open dumps received much of the same waste that MSWLFs receive today. Both open dumps and MWSLFs emit air pollutants through many of the same processes. As a result, open dumps and MSWLFs are both considered throughout this article.

When organic waste decomposes in landfills or open dumps, primarily through bacterial activity, it produces a mixture of gases, including carbon dioxide (CO_2_) and methane (CH_4_) as the main components of “landfill gas”. MSWLFs and open dumps also emit smaller amounts of pollutants like hydrogen sulfide (H_2_S), ammonia (NH_3_), and volatile organic compounds (VOCs) in ambient air [1]. Also, chemicals may form in a landfill or open dump as different waste materials contact and react with each other. For example, chloramines are formed when discarded bleach reacts with NH_3;_ particulate matter is formed through various processes, including oxidation of H_2_S and the sulfur dioxide (SO_2_) to form sulfate particles, and nitrogen oxides can react with newly formed sulfates to produce nitrate-containing particulate matter. Physical processes can emit particulate matter, like decomposing plant litter, the breakdown of plastic waste into microplastics, and the resuspension of silicate dust when trucks travel on dirt roads [1,2,3].

Various pollutants released by MSWLFs and open dumps are toxic. Whether these pollutants might cause harmful health effects among exposed populations depends on many factors, including the following [4]:

- Number of chemicals involved (i.e., a single chemical or a mixture of chemicals).
- Size of landfill and magnitude of emissions.
- Proximity to and susceptibilities of nearby populations.
- Levels of contaminants of concern (i.e., concentration at or above the effect level).
- Environmental conditions (e.g., prevailing wind patterns) [4].

Future studies involving multiple regression analysis can evaluate the contributions of the various factors. Many epidemiological studies have investigated associations between exposure to landfill-related air pollutants and harmful health effects [5,6,7,8,9]. Several review articles have analyzed the published literature [2,3,4,5,6,7,8,9]. These studies indicated that there is limited or inadequate evidence supporting connections between MSWLFs and health effects (e.g., cancer, birth defects, and harmful respiratory outcomes). Most of the studies failed to provide significant evidence of a causal link between MSWLFs and health effects due to a lack of reliable exposure assessment data [10]. Our review is unique because it is based on more than 40 years of ATSDR site-specific assessments of 125 MSWLFs and open dump sites with air pathway evaluations. In addition, we used exposure assessment data that were consistently evaluated or collected using the National Institute for Occupational Safety and Health (NIOSH) Manual of Analytical Methods [11] and U.S. EPA air-monitoring methods [12]. Lessons learned on potential exposures, health impacts, and recommended public health actions were discussed.

### Background on ATSDR Site-Specific Evaluations

Federal law requires the ATSDR to conduct public health assessments (PHAs). PHAs are evaluations for National Priorities List (NPL) sites (commonly referred to as “Superfund” sites). The ATSDR also evaluates sites in response to petitions that the public, elected officials, government agencies, and other parties submit to the agency. These evaluations typically culminate in the ATSDR preparing and issuing a PHA or HC [13].

PHAs and HCs are site-specific assessments that evaluate whether exposures to environmental contamination present a public health hazard (i.e., have the potential to result in harmful health effects in exposed community members). The site-specific documents generally evaluate existing environmental sampling data, which may be collected by site owners, government agencies, academics, community groups, and other parties. In some instances, the ATSDR will collect environmental or biological samples to fill important data gaps. All data considered by the ATSDR are reviewed for quality before being used [13]. The PHA process includes three general steps: initial screening of maximum contaminant data against substance- and media-specific comparison values; estimating site-specific exposures for comparison against health guideline values; and in-depth toxicological evaluation considering how exposures compare to effect levels in the toxicological literature. Additional information can be found in the ATSDR’s online public health assessment guidance manual (PHAGM) at the following link: https://www.atsdr.cdc.gov/pha-guidance/index.html [13].

The ATSDR developed procedures for estimating exposure data and its potential health effects through the PHA process [13]. This is the first time that anyone has comprehensively aggregated and analyzed the MSWLFs’ PHA results and conclusions in a scientific review paper [2,9,10]. The purpose of this article is to summarize what the ATSDR learned from reviewing air pathway evaluations for MSWLFs and open dumps. Air pathway evaluations include assessments of both outdoor air exposures and indoor air exposures (e.g., vapor intrusion of contaminants that originated at MSWLFs). These evaluations consider health hazards resulting from exposure to toxic substances and safety hazards from fire or explosions associated with the unsafe accumulation of flammable gases.

## 2. Materials and Methods

### 2.1. Document Identification

**Figure 1:**
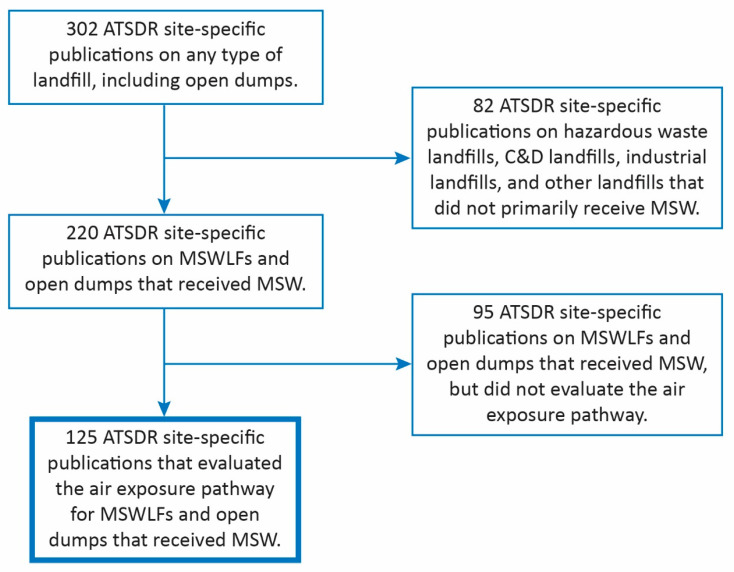
Selection criteria for the ATSDR publications on MSWLFs PHAs, or HCs assessment.

Figure 1 summarizes the process the ATSDR applied to identify PHAs and HCs. First, the ATSDR reviewed its internal files to identify the ATSDR’s PHAs and HCs for landfills or open dump sites. The ATSDR also considered input from state agencies that hold cooperative agreements under the ATSDR’s Partnership to Promote Localized Efforts to Reduce Environmental Exposure (APPLETREE) program. Through this effort, the ATSDR identified 302 PHAs and HCs for consideration. These documents were issued between 1988 and early 2025.

Second, the ATSDR classified these 302 documents by type of waste disposal facility. This step determined that 82 out of the 302 documents were outside the scope of this effort. The 82 documents pertained to hazardous waste landfills, construction and demolition debris landfills, industrial waste landfills, and composting facilities. Those documents were not considered for further assessment. The remaining 220 publications were for MSWLFs or former open dumps.

Third, the ATSDR reviewed the 220 documents to determine which subset considered the air exposure pathway. This step was needed because PHAs and HCs may address different exposure pathways (e.g., inhalation, ingestion, and dermal contact). This assessment focuses on the ATSDR’s evaluations of air emissions from MSWLFs and open dumps. Among the 220 documents that were reviewed at this step, 125 included an ATSDR conclusion pertaining to the air exposure pathway. These 125 documents are examined in this review.

### 2.2. Characterization of the Selected MSWLFs and Open Dumps

The ATSDR developed an Excel database of site characteristics. Fields in the database included various parameters and landfill features expected to affect the magnitude of air emissions. Examples include the following:

- The year of construction;
- The landfill’s size in acres;
- Whether the site was active or closed at the time of the ATSDR’s publication;
- Whether the site had a landfill gas-collection system, and if so, whether it was a passive or active system;
- Whether or not the site had been an open dump;
- Whether the site had ever had a landfill fire;
- Whether redevelopment activities occurred that resulted in public use atop previous disposal sites.

Some documents did not provide enough detail on these factors or did not address them at all. In these cases, the ATSDR classified the information as “unclear” or “missing.”

In most cases, the ATSDR used only information from the PHA or HC to populate site-specific fields in the database. However, when necessary, the ATSDR considered other information sources. For instance, the ATSDR used the EPA’s Landfill Methane Outreach Program (LMOP) database to confirm whether certain sites had landfill gas-collection systems. The LMOP database was also used to determine whether the landfill was active or closed and the type of landfill gas-collection systems used [14].

The ATSDR designated a site as an MSWLF if any of the following criteria were met: (1) the ATSDR publication specifically identified the site as an MSWLF; (2) the EPA listed the site as an MSWLF, either in the agency’s 1992 List of Municipal Solid Waste Landfills or in LMOP resources; or (3) online research clearly indicated that the site was an MSWLF. The remaining sites were considered open dumps. For these, the ATSDR indicated that some portion of the waste received was MSW (e.g., “general household waste”) [1].

### 2.3. Public Health Conclusion Categorization

The final step in the document evaluation was categorizing the public health conclusion for the air exposure pathway. Categories used for this characteristic were as follows: “no conclusion/not evaluated,” “not enough data to evaluate (uncertain public health hazard),” “not a public health hazard,” or “public health hazard.”

The ATSDR’s database also recorded the underlying reasons for a “public health hazard” finding. These fell into three categories: exposures to toxic substances, presence of physical hazards, and exposures to odors. These three categories roughly reflect the types of community concern expressed to the ATSDR. Additional information on determining conclusions of PHAs can be found at PHAGM [13].

### 2.4. Data Analysis

We retrieved documents from the ATSDR’s internal and external databases and organized the details in a Microsoft Excel spreadsheet. Site-specific environmental data and information on health concerns were generally provided by the United States Environmental Protection Agency (EPA), other governmental agencies, businesses, and the public.

The ATSDR developed science-based procedures for estimating exposure and its potential for harmful health effects through the PHA process. Each site-specific assessment included data quality and statistical analysis, such as descriptive statistics. When necessary, the ATSDR recommended further assessments to determine the health hazard category, protect public health, and prevent future exposures or health effects.

## 3. Results

**Figure 2:**
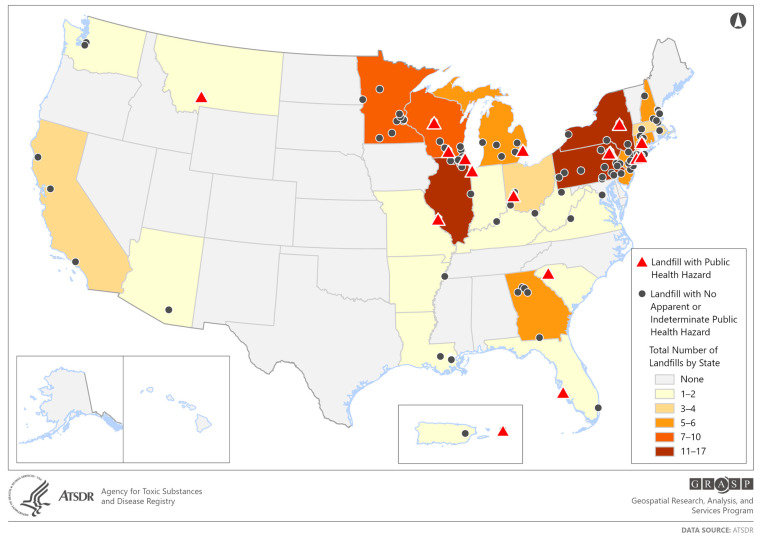
Locations of the 125 MSWLFs and open dumps considered in this analysis.

The ATSDR reviewed findings from 125 publications that addressed air emissions from MSWLFs or open dumps and reached a public health conclusion for the air exposure pathway. Figure 2 shows a map with the locations of the sites evaluated in these documents. While the sites were found throughout the country, a greater proportion were in the Midwest and the Northeastern United States.

Red triangles identify the 18 sites that the ATSDR previously concluded posed a public health hazard through the inhalation pathway. Gray dots depict sites with no apparent or indeterminate public health hazard by inhalation. It is important to note that these 125 documents address a very small portion of the nationwide active and closed MSWLFs and open dumps. In addition, the sites considered in this evaluation are not a representative sample of MSWLFs and open dumps in the United States. Rather, the sites are a subset of MSWLFs and open dumps that are NPL sites or sites for which ATSDR accepted petitions for conducting PHAs.

Table 1 summarizes selected characteristics of the 125 documents. Half of the documents (N = 62) were issued in the 1990s; approximately one-third of the documents (N = 40) were issued in the 2000s; the remainder were issued in the 1980s (N = 2) and the 2010s (N = 21). Most of the documents (N = 100) pertained to MSWLFs and open dumps that were closed and no longer accepting waste; 20 percent of the documents (N = 25) addressed active disposal sites.

Among the 125 documents, 24 percent (N = 30) addressed closed sites that had unrestricted public access to some portions of the disposal surface at the time of the evaluation. Examples of public use include residential developments, office buildings, parks, and walking paths; sites without fencing were also considered in this category. The remaining 76 percent of documents (N = 95) were for sites that, at the time of evaluation, did not have current public uses. Finally, 11 percent (N = 14) of the documents referred to a subsurface fire having occurred at some point during the landfill’s or open dump’s history.

As Table 2 shows, in 107 of the documents, the ATSDR concluded that there was not a public health hazard. This would be for one of two reasons. First, the investigators may have found that the MSWLF or open dump released various toxic substances into the air, but the air concentrations did not result in exposures that would be harmful; thus, it was classified as not a public health hazard. Second, the authors may have concluded that not enough information was available to determine whether a public health hazard existed; thus, the site was classified as an uncertain health hazard. Those documents without public health hazards typically included recommendations to collect additional exposure, health concern information, and information on best practices to eliminate or reduce exposures to safe levels.

In 18 of the documents, in which the ATSDR reached a public health conclusion for the air exposure pathway, the authors reached a conclusion of a “public health hazard.” Table 2 also indicates issues associated with the public health hazard designation: for five sites, the public health hazard conclusion was due to inhalation exposures at sufficiently high enough level to cause harmful effects; for 12 sites, the public health hazard conclusion was due to physical hazards associated with methane gas reaching flammable levels; and at one site, the public health hazard conclusion was due to both toxic substance exposures and methane-related physical hazards.

### 3.1. Public Health Hazards Associated with Inhalation Exposures to Toxic Substances

Table 3 summarizes the landfill characteristics and exposure evaluations for the six sites for which the ATSDR concluded a public health hazard occurred due to inhalation exposure to toxic substances. Below, we discuss different categories of air pollutants.

Reduced sulfur compounds (RSCs): The toxic substances most associated with public health hazards at MSWLFs and former open dumps were sulfur compounds. The compounds include H_2_S, dimethyl sulfide, methyl mercaptan, dimethyl disulfide, and other sulfur compounds such as SO_2_. In this review, RSCs are expressed as H_2_S, as the major component of the RSC mixture. In every case, the public health hazards were due to acute exposures to peak, short-term concentrations of these compounds.

At two NPL sites, the elevated concentrations of sulfur compounds clearly resulted from landfill operational issues. At one site, a subsurface smoldering event (i.e., an underground landfill fire) led to significant settling of previously disposed wastes, and the landfill owner eventually had to reconstruct the site’s leachate and landfill gas-collection systems to reduce the landfill’s air emissions and offsite air quality impacts. During this construction period, offsite H_2_S concentrations averaged for 1 to 3 min reached 3700 ppb. Offsite SO_2_ concentrations for the same averaging time reached 1600 ppb [15,16]. At the second site, the landfill’s leachate collection system experienced operational issues, which resulted in the accumulation of several million gallons of leachate in the landfill. The accumulation resulted in increased H_2_S emissions. Onsite air concentrations of H_2_S reached 3100 ppb, and offsite concentrations exceeded 1000 ppb. The averaging time for these measurements was not specified [17]. In both cases, remediation efforts at the landfills (particularly, upgrades to the leachate and landfill gas-collection systems) reduced the sulfur compound emissions to safe levels.

There were two other cases for MSWLFs constructed in the early 1970s that were not on the NPL. At one of these sites, instantaneous offsite H_2_S concentrations (less than 2 min average) reached 8906 ppb. Instantaneous SO_2_ concentrations (also less than 2 min average) reached 436 ppb [19]. This site was included in this evaluation because it contained two MSWLFs and a construction and demolition (C&D) landfill; however, the isolated and short-lived peak concentrations appeared to have been due primarily to air emissions from the C&D landfill. Subsequent installation and operation of a landfill gas-collection system addressed the issue. At the other site, an instantaneous offsite H_2_S concentration reached 9745 ppb, but the documents provide few additional details to allow further interpretation [20,21].

Volatile organic compounds (VOCs): VOCs were associated with public health hazards at three MSWLF sites. The VOC exposures of concern at two of these sites were due to vapor intrusion. At one site, the VOCs of greatest concern in offsite indoor air samples at nearby residences were benzene, chloroform, and 1,2-dichloroethane [22]. At the other site, an NPL site, the VOCs of greatest concern in offsite indoor air were benzene and vinyl chloride [17]. Both landfill sites share several common features, including the absence of liners, the absence of gas-collection systems, and households being close to the landfill boundary (e.g., homes less than 100 feet away). Vapor intrusion concerns were addressed through landfill remediation efforts like sub-slab depressurization units in homes and the expansion of gas-collection capacity of the landfills. The principal concern in both cases was chronic exposure to carcinogenic benzene and vinyl chloride.

At the third site, methylamine and acetaldehyde had isolated concentrations that reached levels that could have caused odor-induced acute effects. Acute effects include irritation of the eyes, nose, throat, and respiratory tract [20,21]. At each of the three air-monitoring stations for this landfill, methylamine was detected in 1 out of 29 samples, and acetaldehyde was detected in 2 out of 29 samples. The reason for the infrequent and elevated measurements is unclear.

Other air pollutants. The review identified three landfill sites that had public health hazards due to inhalation exposures to toxic substances other than those listed above. First, phosgene, mercury, and combustion byproducts were identified as presenting a public health hazard at a landfill that was experiencing an underground fire and that previously engaged in open burning of some waste [23]. Second, NH_3_ and fine particulate matter (PM_2.5_) were identified as presenting a public health hazard at a different landfill [19,20]. However, the evidence for NH_3_ rested on a single sampling event with suspected data-quality issues and emissions from the landfill, combined with emissions from many other sources contributing to the elevated PM_2.5_ concentrations. Finally, in one case, an HC noted a public health hazard due to observing naturally occurring radon levels, but this hazard was unrelated to the MSWLF being evaluated.

Synthesis: The ATSDR and its cooperative agreement partners identified 125 PHAs and HCs that had air exposure pathway conclusions for MSWLFs or open dumps. For six of the sites (five percent of the sites), the documents identified public health hazards due to concerns regarding inhalation exposure to toxic substances. One should not infer from this statistic that five percent of MSWLFs in the United States have public health hazards due to air emissions of toxic substances. The sites that the ATSDR evaluated are not a representative sample of MSWLFs in the United States. The ATSDR may conduct evaluations at sites with heightened concerns because they involve (a) environmental releases with potential for harmful health effects occurring among neighboring communities, (b) environmental emergency response, or (c) National Priority List (NPL) sites. During the PHA process, environmental data and information on health concerns are obtained from the EPA, the potentially responsible party, or other agencies responsible for conducting environmental investigations. When the available data are of poor quality or there is a data gap, the ATSDR may collect its own data to fill the gap.

Some public health hazards associated with toxic substance exposures were due to outdoor air pollution (most frequently due to sulfur compounds), which resulted from landfill emissions moving through the air to offsite locations. In most cases, the elevated emissions were tied to operational issues, such as landfill fires and a lack of effective landfill gas or leachate management. In nearly every case, the landfill owners addressed the underlying operational issues, which reduced air emissions and eliminated the public health hazard.

Other hazards associated with toxic substance exposures were due to vapor intrusion of migrating landfill gas via the subsurface into offsite residential structures. The vapor intrusion hazards only occurred at unlined landfills with ineffective landfill gas control and where offsite residential structures were in very close proximity (less than 100 feet) to the disposal cells. The ATSDR’s experience at these landfills, which were not constructed according to today’s standards, suggests that indoor air exposures could be reduced to safe levels by actions taken at the landfill (e.g., more effective landfill gas control) and at the affected households, whether through passive mitigation methods (e.g., venting) or active mitigation methods (e.g., sub-slab pressurization).

### 3.2. Public Health Hazards Associated with Methane Gas Reaching Flammable Levels

Table 4 summarizes the landfill gas migration characteristics for the 12 sites that the ATSDR and its cooperative agreement partners concluded had an occurrence of a public health hazard in onsite or offsite structures or in outdoor air due to methane gas at or near a flammable range (5 to 15 percent, by volume). The conclusions regarding methane hazards do not mean fires or explosions occurred. Rather, the PHAs and HCs identified methane concentrations of concern, typically near or above the lower explosive limit (LEL), and noted that fires or explosions could have occurred if the flammable atmosphere met an ignition source.

The sites in Table 4 share several characteristics. First, the MSWLFs and former open dumps that had methane explosion hazards were all older sites: their dates of construction (or initial operation) ranged from 1920 to 1975. Notably, this entire range predates the promulgation of RCRA regulations, indicating that the sites with methane hazards all began accepting waste before important federal regulations applied. Second, 9 out of the 12 open dumps and landfills with methane explosion hazards were NPL sites. This trend partially reflects the fact that the ATSDR’s mandate requires the agency to conduct PHAs for NPL sites, but it is also consistent with expectations, given that NPL sites typically have some amount of offsite contamination. Third, all 12 open dumps and landfills with methane explosion hazards were already closed, and none were actively accepting waste. This finding is also consistent with expectations for two reasons: (a) if the landfills were active at the time the ATSDR evaluated them, they would have likely been subject to current environmental regulations (and may, therefore, not have had methane migration issues) and (b) caps on closed landfills can inhibit methane migration through the surface, resulting in methane gases moving laterally through the subsurface to offsite locations. Fourth, at the time that the ATSDR and its cooperative agreement partners issued the PHAs or HCs, the 12 sites either did not have a landfill gas-collection system or had one that performed poorly. The ATSDR has yet to identify an open dump or MSWLF equipped with an effective landfill gas-collection system that has an offsite methane explosion hazard. Fifth, other factors that contributed to the methane explosion hazards pertained to land use atop and adjacent to former dumps or landfills. At two of the sites, residential structures were already built over the former waste sites or were in the process of being constructed over the former waste sites. Furthermore, the sites with the most serious outcomes pertaining to the methane hazard (i.e., abandonment of homes, small “puff” explosions in basement furnaces) occurred at sites where the nearest residents were 100 feet or less from the landfill property line. The shorter distances between the disposed wastes and the structures likely contributed to the hazards [23,24,25,26,27,28,29,30,31,32,33,34,35,36,37,38].

## 4. Discussion

### Review of Key Findings

This analysis identified some common features and factors at MSWLFs and former open dumps where the ATSDR and its cooperative agreement partners identified public health hazards. Examples included site design features (e.g., lack of liners) not consistent with today’s landfill design standards, lack of properly functioning landfill gas-collection systems, underground fires or smoldering events, redevelopment of former disposal sites for other uses (e.g., using the landfill property for residential or commercial developments), and short distances between landfills and the nearest offsite residents. Currently, we do not know the specific contribution of each identified feature or factor. Further research is needed to estimate the contribution of each factor in predicting public health hazards.

Overall, the majority (86 percent) of MSWLFs and open dumps evaluated by the ATSDR regarding the air exposure pathway did not present a public health hazard. Two potential scenarios can be considered: (i) the MSWLF or open dump released various toxic substances into the air, but the air concentrations did not result in exposures that would be harmful; (ii) or the authors concluded that not enough information was available to determine whether a public health hazard existed. When exposure levels are lower than the health-based guidelines such as ATSDR minimal risk levels (MRLs) [39], the initial considerations were that MSWLFs were located, designed, operated, and maintained in a manner that limited health risks to neighboring communities, even though these sites can contain extremely large volumes of toxic substances. The assessments of these sites were completed and closed with stakeholder-specific recommendations. Moreover, even in cases where these sites presented public health hazards, the ATSDR’s experience suggests that a range of actions can be taken over relatively short time frames to reduce concerning emissions or to monitor for unsafe conditions, thus eliminating or reducing the public health hazards. The results are consistent with various scientific and technological efforts being made by the EPA to protect human health and the environment, including the following:

- Promoting the installation of a gas-collection and control system at existing MSWLFs;
- Using engineered sanitary landfills;
- Using the revised criteria in Title 40 of the Code of Federal Regulations (CFR) part 258, integrations of the final rule technical revisions and clarifications of the national emission standards for hazardous air pollutants (NESHAP) for MSWLFs;
- Implementing the landfill gas energy project/landfill methane outreach program [14].
- Implementing vapor intrusion strategies to eliminate or lower benzene and chlorinated VOCs’ indoor levels (i.e., reported as among the most frequent chemicals of concern for vapor intrusion from the PHAs) [40,41].

Whenever new data becomes available and the public concern is not resolved, the ATSDR, along with federal, state, tribal, and local partners, may re-evaluate the situation. Also, checks on a regular basis on the progress of the recommended public health actions given in the site-specific PHA are performed [13].

Methane-related landfill fires at the surface and subsurface drove the public health hazard concern at 12 MSWFL sites out of 18 in this category, in addition to the ATSDR site-specific PHA or HCs evaluation and the 2001 ATSDR Landfill Gas Primer: An Overview for Environmental Health Professionals (e.g., see Section 3) [1]; very few publications discuss landfill methane gas safety and fire hazards and their prevention and control in MSWLFs [42]. Methane gas is highly flammable and can ignite, causing fires. Methane, at high concentrations (i.e., 50% and higher), may cause asphyxiation in humans (i.e., oxygen levels drop below 16%). As it migrates, it is diluted in the air and accumulates in enclosed spaces. At concentrations between 5% and 15% by volume at room temperature, it can form an explosive mixture with air. Over 14% of methane emitted in the air comes from landfills, and it is a key ingredient in forming ground-level ozone [1,14,42]. Ground-level ozone is a lung irritant; long-term exposure is linked to more severe conditions and increased mortality from both respiratory and cardiovascular causes. Furthermore, methane is a powerful greenhouse gas and contributes to climate change [1,14,42,43].

Limitations of this paper include (i) focusing on emissions released into the air and the inhalation exposure pathways only. The oral exposure pathway (e.g., through contaminated water and food consumption) is the subject of a separate paper under development. (ii) The review of the ATSDR PHA documents cannot be generalized to all MSWLFs nationwide. To improve coverage of the review, we additionally integrated the results of the most recent review papers on municipal solid waste management, environmental emissions, and their public health impacts [6,7,9,44]. Also, the LMOP project and landfill data by state provide a better picture of MSWLFs throughout the nation [14,42]. The growing waste-to-energy conversion in MSWLFs is not discussed well in this review. However, detailed information can be found through the LMOP [14] and recent comprehensive reviews, such as Mukherjee et al. (2020) [45], are progressively growing in the U.S. (iii) The ATSDR PHAs considered in this paper were published between 1988 and 2025, and many things have changed over the years, including the type of landfills (e.g., uncontrolled or dumpsite, and sanitary and green waste landfills), changing sampling methodology, and availability of toxicological information, etc., which means that the conclusions reached in the past would not necessarily be the same conclusions reached today. Published research and EPA work indicate an increasing trend of gas landfill conversion to energy and adoption of sanitary landfills and environmentally sustainable technologies that are known to lower environmental impacts [3,14,43,44,45,46]. EPA and NIOSH sampling and analytical reference methods used for RSCs, VOCs, NH_3_, and trace heavy metals measured from outdoor and indoor air near MSWLFs mostly did not change except for some innovations (e.g., gas chromatographic coupled mass spectrometry; portable instead of on the benchtop instruments). However, the access and availability have increased significantly due to innovations and the rapid growth of direct-reading and sensor technologies [11,12,14].

## 5. Conclusions

In general, our review and other published scientific reviews are consistent with the public health concerns associated with populations living in the vicinity of municipal solid waste landfills. MSWLFs built after 1979 EPA promulgated regulations with criteria for sanitary landfills and ended open dumps, properly siting (e.g., a minimum of 100 feet buffer zone between the landfill and residence property line), installation of landfill gas-collection system, and continuous monitoring of methane levels to control for fire and explosion hazards, and maintaining best practice operations have positively contributed to public health protection. Robust environmental epidemiological methods are needed to both integrate new emission control technologies and better estimate human health risks from air emissions of MSWLFs.

The ATSDR’s recommended public health actions usually include site-specific community environmental health education and online resources. Site-specific assessments and publicly accessible documents are available at the ATSDR’s Public Assessments and Health Consultations website [13]. Furthermore, the ATSDR published chemical-specific toxicological profiles available at https://www.atsdr.cdc.gov/toxicological-profiles/about/index.html (accessed on 17 January 2025) for most of the toxic air substances reported in the MSWLFs’ PHAs considered [2,39].

## Figures and Tables

**Table 1:** Breakdown of the 125 MSWLFs * and open dump sites with air pathway conclusions in ATSDR documents, by selected characteristics.

Data Element	Count	Percentage (%)
Decade PHA ^‡^ or HC ^‡^ was issued.		
1980s	2	1.60
1990s	62	49.6
2000s	40	32.0
2010s ^¶^	21	16.8
Landfill or open dump status at time of evaluation.		
Active	25	22.0
Closed	100	78.0
Public use/access of landfill or open dump surface? ^§^		
Yes	30	20.0
No	95	80.0
According to the PHA/HC, did the site have a subsurface fire?		
Yes	14	11.0
No or not specified	111	89.0

* MSWLFs—Municipal solid waste landfills. ATSDR–Agency for Toxic Substances and Disease Registry. ^‡^ PHA or HC—Public health assessment or health consultation. ^§^ Public access to the landfill or open dump surface applies to closed sites that have undergone redevelopment activities. They may include sites with residential developments, parks, walking paths, or other uses. ^¶^ Landfills assessed in the 2020 to early 2025 period did not include MSWLFs.

**Table 2:** Breakdown of the 125 MSWLFs * and open dump sites with air pathway conclusions by public health hazard conclusion.

Data Element	Count	Percentage (%)
PHAs and HCs for MSWLFs * and open dumps that reached a conclusion for the air exposure pathway.	125	100
Subset of PHAs ^†^ and HCs ^†^ that evaluated the air exposure pathway and found either no or no apparent public health hazard.	107	86
Subset of PHAs ^†^ and HCs ^†^ for which the ATSDR ^‡^ or its public health partner concluded a public health hazard for the air exposure pathway. Further breakdown:	18	14
Public health hazard due to exposure to toxic substances.	5	
Public health hazard due to unsafe methane accumulations.	12	
Public health hazard due to exposure to both toxic substances and unsafe methane accumulations.	1	

* MSWLFs—Municipal solid waste landfills. ^†^ PHA and HC—Public health assessment and health consultation. ^‡^ ATSDR—Agency for Toxic Substances and Disease Registry.

**Table 3:** Overview of sites with public health hazards due to inhalation exposures to toxic substances.

Site	Year Disposal Began	Operational Status	Acres	^§§^ Substances with Public Health Hazard	Description	References and Additional Notes
1	1950s	Closed	52	RSCs and SO_2_	The closed landfill is a former limestone quarry, with mostly municipal solid waste disposed of at the site. It is part of an NPL site. Community concerns about emissions increased after a “subsurface smoldering event” (i.e., underground landfill fire) was detected. This event led to considerable remediation efforts at the landfill. Ambient air-monitoring for RSCs and SO_2_ found levels that might be harmful to health. Exposures to the highest concentrations might have aggravated chronic respiratory disease (e.g., asthma), aggravated chronic cardiopulmonary disease, and caused other respiratory effects (e.g., difficulty breathing, tightness in the chest). These concerns were greatest during the remediation efforts following the landfill fire, and the remedial efforts effectively reduced the landfill’s air quality impacts.	Semkiw et al., 2018, 2022 [15,16]
2	1974	Closed	113	H_2_S and VOCs	The closed landfill is an NPL * site. The location was originally used to mine sand and gravel, before it was used as a construction and demolition debris landfill, and eventually as an MSWLF ^†^. In the early 1990s, the landfill’s leachate collection system did not function properly, and several million gallons of leachate accumulated in a landfill section. This caused increased H_2_S emissions, with fenceline ambient air concentrations greater than 1 ppm—levels that the ATSDR ^‡^ concluded were a public health hazard. Installation of an active landfill gas-venting system and upgrades to the leachate collection system reduced H_2_S concentrations to safe levels. VOCs in indoor air (particularly benzene and vinyl chloride) were a public health hazard, due to vapor intrusion of landfill gases that migrated in the subsurface and into homes, some of which were located 100 feet from the landfill boundary. This hazard was addressed through the installation and operation of an active gas-venting system.	NYSDOH (New York State Department of Health). 1995 [17];
3	1970	Closed	200	Radon and VOCs (Benzene, 1,2,4-Trimethylbenzene, 1,2-Dichloroethane, and 1,2-Trans Dichloroethene).	The closed, unlined landfill accepted municipal solid waste for nearly 40 years. Two residential developments were constructed while the landfill was active, with some homes being located less than 500 feet from the landfill cells. Due to concerns regarding vapor intrusion, indoor air sampling occurred at nearby houses. The public health hazard finding was based on estimated lifetime cancer risks greater than 1-in-10,000 from multiple VOCs (including benzene, chloroform, and 1,2-dichloroethane). The document also noted that exposures to naturally occurring radon contributed to considerably higher cancer risks, though the radon issues were not attributed to landfill releases. Affected homes were equipped with sub-slab depressurization units, which have reduced cancer risks below levels of health concern.	Cit of Bozemana and Tetra Tech 2014 [18]; Elgethun K. 2015 [19]
4	1973	Closed	Not specified	Odors, H_2_S and SO_2_	Multiple landfills operated on adjacent properties, including two MSWLF cells and a construction and demolition (C&D) ^§^ debris landfill. Residences were located less than 100 feet from the C&D landfill cell. Ambient air-monitoring revealed that H_2_S levels were “high enough to cause temporary respiratory discomfort to those with asthma, and perhaps even in some without asthma who suffered from other preexisting respiratory conditions.” One peak SO_2_ concentration reached levels that could have caused clinically significant symptoms among residents with asthma. In response to the air quality impacts, landfill operators installed a gas-collection system that helped prevent landfill gases from reaching nearby residents. The HC focuses on the C&D landfill at the site, but acknowledged that all three adjacent landfills have sulfur compound emissions.	Sutton et al., 2010 [20]
5	1970s	Active	~1000	H_2_S, NH_3_, VOCs (Benzene and formaldehyde, and particulate matter.	Approximately three-fourths of the 7000 tons of waste disposed of daily at the landfill was municipal solid waste, with the remainder being C&D debris, non-hazardous industrial waste, and other non-hazardous waste. Ambient air monitoring conducted in the area found several toxic substances that occasionally reached concentrations that could have caused transitory health effects among sensitive populations, such as irritation to the eyes, nose, throat, and respiratory tract. Substances of concern were NH_3_, H_2_S, methylamine, and acetaldehyde, and only the highest sampling results reached levels of potential concern. Some data-quality concerns were noted for the sampling data. PM_2.5_ concentrations also reached levels of potential health concern on a few sampling dates and when winds blew from the direction of the landfill, but other emission sources also contributed to the airborne levels.	Arunachalam et al., 2019 [21]
6	1979	Active	34	Phosgene, mercury, and combustion byproducts, VOCs including benzene, 1,2,4-Trichloropropane; Chlorobenzene; Acetaldehyde, and metals (e.g., Ni, Hg, As).	The landfill primarily accepts municipal solid waste, but also accepts C&D debris and industrial waste. The ATSDR became involved with the site after residents voiced concern about air emissions from an underground landfill fire, which had been burning for at least several months, according to multiple accounts. The landfill also experienced an aboveground fire in a pile of tires, and the landfill previously had a “burn pit” and “smolder pit” on its property. The nearest residents were approximately 500 feet from the landfill’s active section. Air samples collected on the landfill found concentrations of phosgene and mercury at levels of potential health concern. Elevated levels of several aldehydes were also noted. The public health hazard reported in the PHA was attributed to the combined effect of such respiratory irritants as smoke, various aldehydes, phosgene, and mercury vapor. The principal recommendation made in the PHA was to extinguish the underground landfill fire that was believed to contribute to the public health hazard.	Langmann et al., 1998 [22]

* NPL—National Priority List. ^†^ MSWLF—Municipal solid waste landfills. ^‡^ ATSDR—Agency for Toxic Substances and Disease Registry. ^§^ C&D—Construction and demolition. ^§§^ Chemical substances (RSC—reduced sulfur compound; SO_2_—sulfur dioxide; VOC—volatile organic compound; H_2_S = hydrogen sulfide; NH_3—_ammonia; Metals—e.g., Ni—nickel; Hg—mercury; and As—arsenic.

**Table 4:** Overview of sites with public health hazards due to methane gas reaching flammable levels.

Site	Year Disposal Began	Operational Status	Acres	Description	Reference
1	1974	Closed	10	This unlined landfill, an NPL * site, primarily accepted municipal solid waste, but also received non-hazardous industrial waste (e.g., oily sludge, metal grindings). Soil gas sampling occurred at various onsite locations, with methane concentrations in one portion of the landfill near the main office ranging from 10 to 90 percent. The HC ^†^ refers to an “elevated gas measurement near the landfill office” but does not document the observed methane level. The HC concluded that the potential migration of methane to the landfill office may present a public health hazard. The landfill evidently did not have a landfill gas-collection system at the time the ATSDR ^‡^ evaluated the site.	Morse et al., 2008 [23]
2	1920	Closed	13	This unlined landfill accepted municipal, commercial, and industrial waste for nearly 50 years. The landfill area was then covered with clean fill and developed for commercial, residential, and industrial uses, with no landfill gas-collection system in place. At three commercial properties built atop the former landfill, indoor air concentrations of methane exceeded the lower explosive limit (LEL) ^§^, and occupants raised concerns that landfill gas was entering through foundation cracks and could be ignited. Multiple actions were taken to address this physical hazard: buildings were equipped with continuous methane monitors to detect atmospheric concerns; cracks in building foundations were identified and sealed; and passive vents were installed at multiple landfill locations to release landfill gas into the ambient air.	Pestana et al., 1995; McRae 2005; Rusnak et al., 2006 [24,25,26]
3	1963	Closed	49	This unlined landfill, an NPL site, previously accepted municipal solid waste, C&D debris, and sludge. Multiple residential and commercial properties are located immediately north of the landfill boundary. Even though steps had been taken to reduce the migration of landfill gases, methane concentrations in a floor drain in one of the affected buildings exceeded the LEL on 9 out of 12 sampling days in the late 1990s. Elevated methane levels were also observed in the building floor cracks and sumps. The ATSDR concluded that the observed methane levels in the affected buildings were an urgent public health hazard and recommended that actions be implemented to monitor indoor methane levels, to reduce the methane levels to below 10 percent of the LEL, and to prepare occupants for evacuation if methane levels continue to exceed 10 percent of the LEL.	Baughman et al., 1997; Baughman et al., 1998; Baughman et al., 2004 [27,28,29]
4	1975	Closed	62	Although originally approved as a “private sanitary landfill,” this unlined landfill accepted a wide range of waste (including hazardous waste) for nearly 25 years, until the landfill ceased operating. Passive vents were installed with the intent of collecting landfill gas for reuse, but the project was not completed, and the gas was vented into ambient air without flaring. The methane concentrations at the landfill, an NPL site, were high enough to trigger alarms on the explosimeters used by state agency staff who visited the site. The PHA did not document the methane concentration that triggered the alarms, though many of these meters are typically set to alarm when flammable gas levels exceed 10 percent of the LEL. The methane released from the onsite vents was deemed a public health hazard due to the possibility of trespassers or site visitors encountering flammable atmospheres and the lack of warning signs.	Jackson et al., 2012 [30]
5	1968	Closed	145	The unlined landfill, an NPL site, received municipal solid waste from multiple municipalities for at least 7 years. The landfill was not equipped with a landfill gas-collection system. Methane was detected in soil gas samples at the landfill and its perimeter, but the measured concentrations were not reported in the PHA. The document concluded that the methane gas detections at the site presented a potential explosive hazard.	Smith et al., 1995 [31]
6	1963	Closed	108	This landfill, an NPL site, received municipal and commercial solid waste for 27 years. The landfill included two disposal cells: one lined and the other unlined. While the landfill was still operating, elevated methane concentrations were measured in the basements of two homes, both located less than 100 feet from the site boundary. The local city eventually purchased the homes because the indoor methane concentrations had reached “explosive levels.” Following these events, a landfill gas-collection system was installed and continuously operated to prevent further migration of methane gases. Routine soil gas monitoring has demonstrated the system’s effectiveness. The ATSDR concluded that the site presented a past public health hazard due to the migration of methane gas to nearby households and recommended that the performance of the landfill gas-collection system be continuously monitored to ensure methane gas does not migrate offsite at unsafe levels in the future.	Crua et al., 1996 [32]
7	1947	Closed	34	This unlined landfill, an NPL site, received municipal, commercial, and industrial waste for more than 40 years before closure. Two studies (a soil gas survey and a methane migration study) examined the potential for methane gas to migrate offsite at explosive levels. The soil gas survey identified four methane “hot spots” at the landfill, and the migration study reported elevated methane concentrations at a neighboring property. Another study detected methane concentrations above the LEL on three different occasions at a location outside of the landfill property. These observations contributed to the finding of a potential explosion hazard. Proposed remediation measures for the landfill were to include monitoring and controlling methane migration, but further details on the specific remediation measures were not specified.	Raffert et al., 1995 [33]
8	1974	Closed	113	This unlined landfill, an NPL site, primarily received municipal solid waste for 23 years. Migration of landfill gas to offsite locations was extensively documented. Methane concentrations greater than the LEL were measured in several homes within 100 to 500 feet of the landfill, and methane concentrations greater than 50 percent of the LEL were measured in buildings in a nearby industrial park. The elevated methane concentrations caused “furnace puff-backs” at several homes, indicating explosive levels of methane. Concern about methane migration heightened in the winter months when surface soils were often frozen. The landfill operators eventually installed a series of active and passive gas vents, some of which became part of the “landfill perimeter gas-collection system.” The ATSDR recommended development of an operations and maintenance plan for the collection system, given its critical role in ensuring landfill gas does not cause unsafe methane accumulations in offsite structures.	Schuck et al., 1995 [34]
9	1966	Closed	100	This NPL site comprises multiple former quarries that were used for disposal of municipal, commercial, and industrial solid waste over a 23-year period starting in 1966. Soil gas samples were collected around the perimeter of the landfill, and methane concentrations exceeded the LEL at multiple locations, including within 200 feet of high-density residential areas. Methane soil gas concentrations above the LEL at the landfill perimeter were confirmed in multiple rounds of additional sampling. These observations led to a conclusion of a public health hazard, which was to be addressed through several actions. These include the installation of a network of passive vents around the landfill perimeter and the operation of combustible gas monitors inside the homes nearest to the landfill.	ODH and ATSDR 1998 [35]
10	1972	Closed	4	This unfenced and unlined open dump accepted municipal and industrial waste for 6 months. The waste was eventually covered with soil, but no access restrictions were in place, and a home was being built atop a former disposal area at the time the site was assessed. Gas-monitoring probes placed around the perimeter of the landfill found methane concentrations above the LEL at two locations. Subsequently, monthly monitoring of landfill gas had multiple measurements of methane gas above the LEL and additional measurements marginally below the LEL. Methane concentrations also exceeded the LEL at a crack in the dump surface. The HC conclusion was that the site presented a future public health hazard due to the risk of explosion, should methane gases accumulate in the crawl space of the home that was under construction.	Shelley et al., 2004 [36]
11	1970	Closed	80	This unlined landfill, an NPL site, received municipal and industrial waste for 5 years before being closed. Following the detection of methane gas in a groundwater monitoring well, a soil gas-monitoring network was deployed to determine the nature and extent of methane migration away from the landfill property. At four soil gas-monitoring sites along the site perimeter, methane concentrations exceeded the LEL on multiple occasions. Methane gas monitoring in nearby homes (all within 500 feet of the site) had not found elevated concentrations, but the site was considered a physical hazard due to the potential for further gas migration to occur.	Nehls-Lowe et al., 1994 [37]
12	1952	Closed	27	For 30 years, this unlined dump, an NPL site, received municipal, industrial, and C&D waste. During site investigations, elevated methane concentrations were detected at a monitoring well. A comprehensive methane survey followed, in which soil gas methane concentrations above the LEL were identified in multiple onsite areas, including under an onsite building. The nearest homes (approximately 1000 feet from the dump) were also monitored for methane gas, but the measured concentrations were not elevated. The document concluded that the potential buildup of methane gas beneath structures at the dump was an explosion hazard.	Goldrina et al., 1994 [38]

* NPL—National Priority List. ^†^ HC—Health consultation. ^‡^ ATSDR—Agency for Toxic Substances and Disease Registry. ^§^ LEL—Lower explosive limit.

## Data Availability

PHA documents were the major data sources. All PHAs reviewed were released and available to the public.
